# A Theory of Predictive Dissonance: Predictive Processing Presents a New Take on Cognitive Dissonance

**DOI:** 10.3389/fpsyg.2018.02218

**Published:** 2018-11-19

**Authors:** Roope Oskari Kaaronen

**Affiliations:** Department of Social Research, Faculty of Social Sciences, University of Helsinki, Helsinki, Finland

**Keywords:** predictive processing, predictive coding, cognitive dissonance, affordance theory, ecological rationality, cultural evolution

## Abstract

This article is a comparative study between predictive processing (PP, or predictive coding) and cognitive dissonance (CD) theory. The theory of CD, one of the most influential and extensively studied theories in social psychology, is shown to be highly compatible with recent developments in PP. This is particularly evident in the notion that both theories deal with strategies to reduce perceived error signals. However, reasons exist to update the theory of CD to one of “predictive dissonance.” First, the hierarchical PP framework can be helpful in understanding varying nested levels of CD. If dissonance arises from a cascade of downstream and lateral predictions and consequent prediction errors, dissonance can exist at a multitude of scales, all the way up from sensory perception to higher order cognitions. This helps understand the previously problematic dichotomy between “dissonant cognitive relations” and “dissonant psychological states,” which are part of the same perception-action process while still hierarchically distinct. Second, since PP is action-oriented, it can be read to support recent action-based models of CD. Third, PP can potentially help us understand the recently speculated evolutionary origins of CD. Here, the argument is that responses to CD can instill meta-learning which serves to prevent the overfitting of generative models to ephemeral local conditions. This can increase action-oriented ecological rationality and enhanced capabilities to interact with a rich landscape of affordances. The downside is that in today’s world where social institutions such as science *a priori* separate noise from signal, some reactions to predictive dissonance might propagate ecologically unsound (underfitted, confirmation-biased) mental models such as climate denialism.

## Introduction

Cognitive dissonance (CD) theory is arguably one of the most influential and extensively studied theories in social psychology. Dissonance theory continues even 60 years after its original formulation by Festinger (1957), in *A Theory of Cognitive Dissonance*, to develop and inspire new research (Cooper, 2007; Harmon-Jones et al., 2015). However, it is sensible to attempt to bring even the most influential and mature theories up to date with current scientific advances. The present article aims to do precisely that by integrating insights from CD theory and predictive processing (PP, also known as predictive coding). In this article, I accommodate dissonance theory in the broader framework of PP, and call this synthesis “predictive dissonance.” PP, a unificatory attempt at understanding action, perception, and learning, has already found multiple applications in cognitive science, machine learning, and brain theories (for overviews, see Friston, 2010; Clark, 2016), but its application to traditionally more social psychological domains still seems scarce (although see Ramstead et al., 2016). I suggest in the present article that dissonance theory – in its original form (Festinger, 1957) as well as its more recent “action-based” (Harmon-Jones et al., 2015) and evolutionary (Egan et al., 2007) propositions – is highly compatible with PP, and a comparative take on the two theories can be mutually informative. This article is therefore an attempt to reconcile a classic theory (Brehm, 1956; Festinger, 1957; Festinger and Carlsmith, 1959) with a novel take on cognitive processes.

The rationale for writing such a comparative study is not to “explain away” the theory of CD with a newer theoretical framework, but rather to provide fresh perspectives for interpreting *what* CD is (related to the concept of “prediction error” in PP), *where* it is (located at varying hierarchical steps of an action-oriented multi-level generative model) and *why* such a cognitive phenomenon might exist to begin with (it can motivate ecologically rational meta-learning, preventing the overfitting of cognitive models). The present study is therefore perhaps best understood as providing evidential diversity (Kuorikoski and Marchionni, 2016) for interpreting a widely known and studied cognitive phenomenon. As this article concludes, CD is, as a mental and social phenomenon, as important a research topic as ever. Furthermore, Festinger (1957) original work was, even in the light of new advances, quite well on track, and its basic premises should not be dismissed. However, bringing the theory of CD up to date with recent developments in embodied and situated cognitive science, particularly by associating our motivation for “dissonance reduction” (Festinger, 1957) with “prediction error reduction” (Friston, 2010; Clark, 2016) provides a clearer picture of what CD is, why it exists, and how and why it structures our lives and cultures so thoroughly.

The present text is structured as follows. Section “Predictive Processing: A Brief Overview” presents a brief introduction to PP. Section “Predictive Dissonance,” in turn, presents the theory of CD in a nutshell and proceeds to a comparative analysis and synthesis between PP and CD theory. Section “An Evolutionary Rationale for Cognitive Dissonance?” presents a potential PP-informed evolutionary rationale regarding the origins of CD. Section “Underfitted Generative Models and Ecological Crises” briefly discusses some current ecologically unsound forms of CD (related to, e.g., climate denialism) and discusses these in terms of the propositions made in earlier sections. Section “Conclusion” summarizes the core arguments of this article.

## Predictive Processing: a Brief Overview

Predictive processing, many of its proponents (e.g., Friston, 2010; Seth, 2014; Clark, 2015, 2016; Allen and Friston, 2018) argue, is a particularly promising unificatory account of perception, action, experience, and expectation, thereby providing a theoretical (and increasingly empirical) account of a fundamentally embodied and environmentally situated mind. In this section, I introduce the main tenets of PP, particularly drawing on Clark’s (2016) work, acknowledging that any brief introduction to such a multifaceted, multidisciplinary, and multilevel account of the mind will be incomplete. Note also, as Clark (2016, Ch. 10) himself emphasizes, this is certainly not the only plausible account of PP, otherwise often known as “(hierarchical) predictive coding.” Therefore, this section attempts to describe particularly the aspects of PP relevant for CD theory, which in turn is defined and compared to PP in Section “Predictive Dissonance” below. The reader is humbly directed particularly to Clark (2015, 2016) and Friston (2010) for a greatly more comprehensive introductory account to PP.

At its core, PP consists of two main features. First, perception actively involves the use of a Bayesian^1^ hierarchy of acquired prior^2^ knowledge, which serves to predict online the incoming sensory barrage. Thus, from the PP perspective, the brain is “an inference machine,” which is in the business of actively predicting and explaining its sensations (Friston, 2010, p. 129). This predictive faculty is known as the multi-level hierarchical “generative model” (ibid.). In lay terms, the PP narrative of embodied and situated cognition entails that the brain is a “predictive engine,” actively predicting sensory input, contrasting these “downstream” predictions to “upstream” sensory information. This stands against what Clark (2015, p. 5) describes as “standard (passive, feedforward)” images of sensory processing, where the brain is perceived “as passive and stimulus-driven, taking energetic inputs from the senses and turning them into a coherent percept by a kind of step-wise build-up moving from the simplest features to the more complex.” Instead, PP proposes a “Bayesian flip”: the predictive brain predicts incoming low-level sensory cues from the best prior models of what is likely to be out there (ibid.). With a simple Bayesian twist, perceptual content is then determined by the hypothesis that generates the best predictions – or in Bayesian terms, the hypothesis with the highest posterior probability (Hohwy et al., 2008, p. 688). The cascades of downstream models are, of course, experience- and context-laden in that they are sculpted by prior experience and contextual factors (see particularly Ch. 9 in Clark, 2016).

Second, the use of prior knowledge – which primarily serves a predictive function – is, according to Clark (2015, p. 5) “subject to a constant kind of second-order assessment (known as “precision estimation”),” which determines weightings assigned to specific predictions at all levels of information-processing (from direct sensory inputs to higher order cognition) as well as to different aspects of incoming sensory signals. In other words, different weightings are assigned to *prediction errors*, accordingly with the estimated reliability (inverse variance) of the incoming sensory barrage. Highly weighted, or highly reliable, prediction errors are more likely to update the generative model (and *vice versa*). By means of updating the generative model, organisms are, in the long term, capable of making the best possible guesses about the true causes of sensory stimuli and thus inferring the external causes that produce sensory data (Friston, 2010).

**FIGURE 1:**
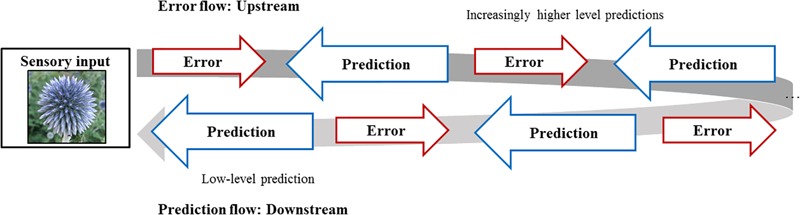
Sensory flow of prediction and perception. In the hierarchical generative model, prediction errors flow upstream (and laterally), and predictions flow downstream.

What results is a dynamic picture of a mind constantly forming generative, forward-looking hypotheses, which are tested against sensory input, which in turn may or may not update the generative predictive model (depending on the weightings assigned to prediction errors). To slightly complicate the picture – which, it should be noted, can be complicated quite a lot further – the Bayesian narrative of the mind proposed by PP suggests that generative models are *hierarchical*, or multi-level predictive systems consisting of nested hypotheses within hypotheses (Figure 1). These hierarchies range from low-level predictions (“often highly spatially/temporally precise”) to increasingly abstract and generalizable high-level predictions (Clark, 2016, p. 30). Prediction errors, within this hierarchical network, flow upstream and laterally,^3^ thereby adjusting generative predictions (which again might result in further unpredicted input, sending error signals to even higher level predictions, see Figure 1) (Clark, 2016, p. 143–146).

The upshot is that the intricate balancing of predictions and prediction errors helps organisms such as ours not only to learn from sensory input but also *not* to learn from low-weighted prediction errors. Therefore, Clark (2016, p. 35; emphasis added) writes, the “task of the predictive brain is to account for (*to accommodate or “explain away”*) the incoming or “driving” sensory signal by means of a matching top-down prediction.” Note that, importantly, the sensory signal can be reacted to in various ways, ranging from the updating of the predictive model, suppressing the error signal, recruiting alternate hypotheses, or altering movement in the world so as to harvest different information from the environment (this is also known as “selective sampling,” ibid.). A reader acquainted with CD theory might predict where this argument is heading – I will return to these alternative strategies for coping with error signals in broader detail and contrast them with dissonance reduction mechanisms in Section “A Comparative Take: Prediction Error and Dissonance Reduction.”

The “predictive engine” of the brain is, according to the PP narrative, also the fundamental driver for action. This is because the brain also makes active forward-looking predictive models for proprioceptive states (i.e., states related to bodily movement and position). If organisms make forward predictions of proprioceptive states, reducing the signal error for these predictions implies moving bodily parts to predicted locations (see particularly Ch 4. in Clark, 2016). Predictions for movement are, it follows, “self-fulfilling prophecies” (Clark, 2016, p. 111).^4^ For instance, expecting the sensory flows of what it would be like if you were to write an academic article on a keyboard, is what (for good or ill) drives one to do so, since any other bodily state would induce prediction error.

The long-term reduction of prediction error, the PP narrative continues, is essential for an organism’s survival in the world. Ultimately, this boils down to the notion that by minimizing prediction errors in the long term, organisms are better equipped to avoid surprising environmental states and encounters which risk their survival. Friston (2010) elaborates this idea much more in his free energy principle, which argues that organisms must avoid surprising states to maintain the far-from equilibrium state of self-organization which we call life (see also Friston and Stephan, 2007; Clark, 2017b; Allen and Friston, 2018; Appendix 2 in Clark, 2016). This is a complex information theoretic and thermodynamic argument which will not be advanced further in the present text, but in lay terms, it simply entails that an organism is more likely to survive in a world it can predict as well as possible (Clark, 2017b, pp. 4–5). The implication is that the predictive organism’s generative cognitive models are, in this respect, locally imperfect but highly generalizable models of the world^5^ (Clark, 2016). Equipped with such models, organisms successfully predict incoming sensory inputs to infer and learn about the causal structures of the external world, all the while avoiding unpleasant and hazardous surprises.

A picture emerges of the predictive mind as one fundamentally driven to guide successful behavior by actively minimizing prediction error. Accordingly, Clark (2016, p. 295) writes: “The predictive brain is thus not an insulated inference engine ‘in the head’ so much as an action-oriented engagement engine, delivering a rolling grip on task-salient opportunities.” Since humans live in complex socio-cultural and material environments, any inference engine of this sort must be capable of adapting to a broad variety of contexts. This notion and its possible implications for understanding CD are further discussed in the latter half of the present article.

## Predictive Dissonance

“When dissonance is aroused, the fun begins in figuring out the best way to reduce it.”

Cooper (2007, p. 109)

To the author’s best knowledge, comparative takes on PP and CD theory, a long-term staple, and dominant paradigm particularly in social psychological research (Cooper, 2007), are either non-existent or lacking. Arguably, to reconcile the theory of CD in the light of recent developments in multidisciplinary studies in PP is insightful in two domains. First, it provides a case that dissonance theory, even though updatable, is still in many respects on track with cutting-edge research even six decades after its initial formulation by Festinger (1957). This is particularly the case since CD theory and PP both deal extensively with the reduction of subjectively surprising states, or dissonance and prediction error, respectively. Indeed, as is shown below, prediction error and CD appear to be processually related, if not at times parallel, phenomena.

Second, a PP take on CD can, I argue, further enlighten recent developments in “action-based” dissonance studies (Harmon-Jones et al., 2015) and help us understand some of the evolutionary underpinnings of CD. These are relevant topics for clarification, since there seems to exist, one the one hand, accounts promoting the idea of dissonance having evolutionary origins (Egan et al., 2007), while others (Perlovsky, 2013) altogether question the compatibility between human evolution and dissonance theory. Moreover, reconciling CD theory with PP – a multidisciplinary theory encompassing machine learning, neural networks, embodied and enactive cognitive science, and ecological psychology – is helpful in relating the phenomenon of CD to a variety of other rapidly developing fields potentially relevant for dissonance research. To begin this comparative treatise, in the following section, I provide a brief overview of the theory of CD.

### Dissonance in a Nutshell

Festinger (1957), in his seminal work *A Theory of Cognitive Dissonance*, defines CD as the psychological discomfort occurring when one experiences two mutually inconsistent “elements of cognition.” Cognitive elements are for Festinger (1957, p. 9–10) what a “person knows about himself, about his behavior, and about his surroundings.” These cognitive elements not only “represent knowledge about oneself” (feelings, pains, desires, attitudes, etc.) but also represent environmental and causal states, concerning “the world in which one lives” or “what is where, what leads to what,” and so forth. While Festinger’s definition of cognitive elements is vague – which is understandable, as Festinger’s pioneering work was conducted at the dawn of the cognitive revolution in psychology – it is clear from his definition that “relations of consonance and dissonance” can hold between all pairs of these elements (ibid.).

For Festinger (1957, p. 10–11), these elements of cognition form “by and large” a “mirror, or map,” of reality. Therefore, it follows that keeping this map or mirror “responsive to reality” – by means of actively reducing dissonance – aids us in maintaining a grip on and surviving in the world (ibid.). Accordingly, Festinger (1957, p. 11) concludes that the major point of dissonance theory “is that *the reality which impinges on a person will exert pressures in the direction of bringing the appropriate cognitive elements into correspondence with that reality*.” Festinger (1957, p. 3) emphasizes that CD is “a motivating factor in its own right,” and that the “existence of dissonance, being psychologically uncomfortable, will motivate the person to try to reduce the dissonance and achieve consonance.” Dissonance reduction and avoidance, in turn, according to the original formulation of dissonance theory, can be achieved *via* four strategies as follows.

#### 1 Changing a Behavioral Cognitive Element

Dissonance can be reduced by changing behavior (Festinger, 1957, p. 19). This is for Festinger (ibid.) the “simplest and easiest way” of reducing dissonance. To use Festinger’s example, if one experiences dissonance when smoking (since she or he knows that smoking is unhealthy), dissonance can be reduced by quitting the smoking habit. Or, to use another simple example of Festinger’s (ibid.), if “a person starts out on a picnic and notices that is has begun to rain, he may very well turn around and go home.”

#### 2 Changing an Environmental Cognitive Element

Dissonance can be reduced by modulating the environment or moving about in the material or social world (Festinger, 1957, p. 20). To illustrate the former, Festinger (ibid.) invites the reader to imagine a man pacing in his living room at home, fearfully jumping over a particular spot on the floor for no valid reason. This induces CD, since the man is well aware that there is no reason to fear this particular spot. Festinger (ibid.) notes that the man could, however, reduce this dissonance by breaking a hole in the floor in that particular place. By modulating his material environment, the man could therefore reduce CD. Festinger (ibid.) calls the modulation of physical environments a “relatively rare occurrence,” a notion which I will challenge in Section “An Evolutionary Rationale for Cognitive Dissonance?,” since humanity as a whole is in fact very active in constructing their cultural and material niche.
Changing social environments, Festinger (ibid.) argues, is a more common affair. For instance, “a person who is habitually very hostile toward other people may surround himself with persons who provoke hostility.” We can also imagine the aforementioned smoker seeking toward environments (e.g., a smoking room) where she or he would experience less dissonance. Importantly, Festinger does not imagine here a passive organism, but rather evokes the idea that we can actively move about in the world to reduce dissonance, for instance, by selecting the social groups we interact with (ibid.). Changing environmental cognitive elements can therefore also be achieved by moving to a different social or physical context.

#### 3 Adding New Cognitive Elements

Festinger (1957, p. 21–24) emphasizes that even if dissonance cannot be eliminated, it can be reduced by “adding new cognitive elements.” Three interrelated sub-strategies emerge here as follows.

##### a Focusing on Supportive Beliefs

Dissonance can be reduced by focusing on more supportive beliefs or reducing the importance of conflicting beliefs. A person might “actively seek new information that would reduce the total dissonance” (Festinger, 1957, p. 22). Thus, a smoker might “seek out and avidly read any material critical of the research which purported to show that smoking was bad for one’s health” (ibid.).

##### b Adding Reconciliatory Cognitive Elements

Dissonance can be reduced by adding cognitive elements or hypotheses which “reconcile” two dissonant cognitive elements. A smoker, Festinger (ibid.) notes, could “find out all about accidents and death rates in automobiles,” reducing the smoker’s dissonance by discounting it against the “more severe” risks of driving. This is, essentially, what dissonance researchers have later called self-justification (see Tavris and Aronson, 2008).

##### c Changing Conflictive Cognitive Elements

Dissonance can be reduced by even arbitrary changes in beliefs, attitudes, or values. Therefore, finally, a smoker might reconsider their belief about the nature of what is “good” or correct behavior (see Festinger, 1957, p. 22–23). In other words, finally, the smoker could conclude that “I simply enjoy smoking and therefore, as I consider it a good behavior, I shall continue to do so.”

#### 4 Avoidance of Dissonance

Next to dissonance reduction, Festinger (1957, p. 29–31) emphasizes that there are also strong tendencies to “avoid the occurrence of dissonance altogether.” In other words, sometimes people will “actively avoid situations and information which would likely increase the dissonance” (Festinger, 1957, p. 3). If a person has past experience that a certain environment or source of information would cause CD, such environments or information might be actively avoided (Festinger, 1957, p. 30).

I have presented above the main tenets of the original composition of dissonance theory (see particularly Ch. 1 in Festinger, 1957, p. 1–31). More recent developments of dissonance theory, such as its action-based model, are discussed later in the present article. One notion worth emphasizing here is the plurality of strategies Festinger (1957) proposes for dissonance reduction, which include not only attitude change but also the alteration of behaviors and environments, movement and interaction in the socio-material realm, and active avoidance of dissonant information. Although dissonance research, since Festinger’s seminal work, has largely focused on dissonance between attitudes or values (Cooper, 2007), I wish to underscore here that this restrictive position is not one found in Festinger’s original theory. Indeed, the focus in dissonance research on attitudinal change is more a product of empirical methodologies, since attitude change (as revealed by, e.g., surveys or interviews) is by many considered the simplest method to empirically study the effects of CD (see Cooper, 2007 for a history of dissonance research).

### A Comparative Take: Prediction Error and Dissonance Reduction

In the following section, I discuss relevant aspects of PP with respect to CD theory. I particularly focus on how many dissonance reduction strategies seem very similar to those found in prediction error minimization. Therefore, in the following section, I explicitly relate Festinger (1957) modes of dissonance reduction (numbered 1–4 above) to PP and prediction error related concepts. This comparative analysis reveals that PP and CD theory are notably similar, and that locating CD in the broader theoretical framework of PP can be mutually informative for both theories.

#### Changing a Behavioral Cognitive Element

Predictive processing posits that prediction error can be reduced by “performing actions that make our predictions come true” (Clark, 2016, p. 121). However, note that with the PP framework, this error reduction mechanism is not merely a reaction to psychological discomfort (although in certain situations it likely is). Rather, PP posits that action itself occurs when “physical motion cancels out prediction errors by producing the trajectory that yields some predicted sequence of proprioceptive states.” This “active inference” (Friston, 2009; Adams et al., 2013; Friston et al., 2016) or “action-oriented PP” (Clark, 2016, p. 122) suggests that percepts are ongoing attempts to “parse the world in ways apt for the engagement of that world.” Prediction error reduction, it follows, is action-oriented, and action is one fundamental strategy to minimize prediction error. This is a particularly relevant notion to consider, since recent developments in CD theory have also suggested an action-based model of dissonance reduction, where it is proposed that dissonance arises particularly with “cognitions with action implications are in conflict with each other, making it difficult to act” (Harmon-Jones et al., 2015). The action-based model of dissonance is returned to in greater detail in Section “Why #1: An Action-Based Model of Dissonance.”

This idea of reducing dissonance in order to be in better “touch with reality” behaviorally is something Festinger (1957, p. 10–11) was certain to emphasize, although it has escaped much of later dissonance research (see Cooper, 2007 for an overview). However, the similarities between dissonance theory and PP, in this respect, are clear. To return to Festinger (1957, p. 19) simple analogy, we could imagine the picnicker experiencing “prediction error” after noticing the rain and reducing this prediction error by moving to shelter. CD and prediction error seem remarkably similar in the behavioral domain, and this theme is discussed in much more detail in Section “An Evolutionary Rationale for Cognitive Dissonance?.”

#### Changing an Environmental Cognitive Element

As with CD, prediction error can also be reduced by two distinct but related strategies, which are the physical modulation of environments and selective sampling of sensory environments (Clark, 2016). First, as is elaborated in detail in Section “Why #2: Overfitting Predictive Models,” humans are organisms which are particularly adept at constructing their environments in order to reduce collective prediction error (Clark, 2016, particularly Ch. 9). Indeed, Clark (2016, p. 270) writes: “We humans … build, and repeatedly rebuild, the social, linguistic, and technological worlds whose regularities then become reflected in the generative models making the predictions.” In other words, we have a tendency for building environments which reduce prediction error. While Festinger (1957, p. 20) did note that CD can be reduced by altering the physical environment, he called it a “relatively rare occurrence.” The predictive dissonance account elaborated in Section “An Evolutionary Rationale for Cognitive Dissonance?” aims to show that environmental modulation, otherwise known as niche construction^6^ (or the process by which an organism alters its local environment), is much more common than Festinger imagined. Nevertheless, recall how Festinger’s living room pacer reduced dissonance by breaking a hole in the floor. This could be easily reformulated as reduction of prediction error. If the man is actively attempting to predict a hole in the ground, while acting as if this were the case, the exteroceptive sensory information he encounters – the stimuli picked up from the external environment, or a perfectly intact floor – would induce upstream flows of prediction error. This prediction error, in turn, can be reduced by the act of breaking a hole in the floor.

Second, similar to how Festinger (1957, p. 20) emphasizes the active role of humans in moving around social environments to reduce dissonance, Clark (2016, p. 70–71) also underscores that in order to reduce prediction error, we actively move about to *selectively sample* the perceivable world. In other words, we have a tendency for selectively harvesting sensory information which is consonant with our predictions. The “dark side” (Clark, 2016, p. 71) of this, confirmation bias, is discussed in more detail later in this section. However, it is worth noting that this sort of confirmation bias was particularly what Festinger, a social psychologist, was interested in. For instance, Festinger (1957, p. 177) emphasizes that dissonance is often most successfully reduced by socializing with people who “agree with the cognitions one wishes to retain and maintain.” Summing up, humans alter environmental cognitive elements constantly, and this is not only a matter of modulating the material environment but also one of moving about in the social and material world so as to actively select the sensory information we harvest.

#### Adding New Cognitive Elements

Similar to propositions from dissonance theory, the Bayesian generative models of PP are active in “finding the predictions that best accommodate the current sensory inputs” (Clark, 2016, p. 121).

##### Focusing on Supportive Beliefs

In the hierarchical PP framework, the recruitment of high-level predictions can explain prediction errors away and effectively tell the upstream-flowing error units to “shut up” (Friston, 2005, p. 829; also Clark, 2016, p. 38). Therefore, lower level inputs which are well predicted by some higher level predictive model are “quashed” or “explained away” (Clark, 2016, p. 60). Moreover, PP proposes that conversely, by increasing *attention*, the gain of error units can be increased. This is called “salience-based signal enhancement” (ibid.). This leads to the boosting of prediction error signals which are deemed reliable, which in turn inform (or update) the predictive generative model, resulting in a very basic form of Bayesian learning. Basically, the suppression or enhancement of prediction error units serves the function of either updating or not updating the Bayesian priors of the predictive generative model.

Note that while this tuning of prediction errors is a very efficient way of sampling the world, it is also prone to result in “*mutually misleading*” cycles (Clark, 2016, pp. 71–72). This is quite fundamental for understanding some of the more problematic aspects of CD, particularly confirmation bias and focus on supportive beliefs. Here, in particular, the precision weighting dynamics of PP can help social psychologists uncover the roots of confirmation biases and means of self-justification associated with CD reduction (see, e.g., Tavris and Aronson, 2008). Clark (2016, pp. 71–72) cites Siegel (2012) notion of perceptual justification to enlighten the “dark side” of prediction error reduction. In this scenario, Jill predicts that Jack is angry at her, leading Jill to selectively sample her perceived visual sensory input to find visual “evidence” for her anger. This in turn, increases the posterior belief of the predictive hypothesis of “Jack is angry,” leading effectively to “a worrying recipe for self-fulfilling psycho-social knots and tangles” (Clark, 2016, p. 75). This is not hard to relate to, for instance, climate denialism, where climate denialists actively sample their information environment to find further evidence for their prior beliefs (see, e.g., Lorenzoni et al., 2007). A similar focus on supportive beliefs can be found in Festinger (1957, p. 22) example of the smoker who actively focuses on information which is supportive toward her or his behavior, or in other words, information which is well predicted by the generative model. Here too, dissonance avoidance appears very similar to active prediction error reduction.

##### Adding Reconciliatory Cognitive Elements and Changing Conflictive Cognitive Elements

A core tenet of PP is that prediction error can be reduced by recruiting “new and better” predictions or by modulating current ones so that they best accommodate current error signals (Friston, 2010; Clark, 2016, p. 1). This compares with, in a way, Festinger (1957) notion that cognitive elements can be added or changed to reconcile dissonant elements. In practice, in the predictive hierarchy, this can also imply that “higher level” hypotheses are saved by adjusting or recruiting new predictions at lower hierarchical levels. Practically, therefore, this could involve, for instance, the arbitrary attitude changes often associated with particular reactions to CD (Festinger and Carlsmith, 1959; Cooper, 2007; Tavris and Aronson, 2008).

To exemplify such arbitrary attitude changes, it is helpful to return to the origins of dissonance theory, which began with a study on a cult known as the Seekers (Festinger et al., 1956; Festinger, 1957, Ch. 10, see also Ch. 1 in Cooper, 2007 for a summary). The original story follows, with PP interpretations in brackets:

Festinger and his students documented a cult, “a serious group” with strong premonitions [generative models] that the world would end by a cataclysmic flood on December 21, 1955. The cultists believed that they could be saved by unearthly beings from the planet Clarion. The Seekers were so certain of their premonitions [high-level prediction: “the world is going to end”] that they sold their possessions and quit their jobs. Yet at the day of the predicted event, with no flood at sight, the Seekers were faced with a calamitous inconsistency [prediction error]. Initially, after midnight, the Seekers sought alternate explanations [recruitment of arbitrary lower-level hypotheses], such as “some of us are wearing items prohibited by Clarions,” which could explain away the inconsistency. Eventually, the inconsistency [prediction error] was diminished by an irrefutable message [recruitment of alternative hypotheses] by the cult leader: “This little group, sitting all night long, has spread so much goodness and light that the God of the Universe spared the Earth from destruction.” Prediction error – or dissonance – was attenuated by saving the high-level hypothesis by recruiting consonant low-level hypotheses. Funnily enough, the title of Festinger et al. (1956) book, “When Prophecy Fails,” could therefore be as rephrased as “When *Prediction* Fails.”

Moreover, PP can help us further understand the “magnitude” of dissonance, which was a central concept in Festinger (1957) original proposition of dissonance theory, although it has since received less attention (Beauvois and Joule, 1996). Formalizing the idea of magnitude, we imagine here the

“sum of all the dissonances involving some particular cognition as ‘D’ and the sum of all the consonances as ‘C.’ Then we might think of the total magnitude of dissonance as being a function of ‘D’ divided by ‘D’ plus ‘C.”’ (Festinger and Carlsmith, 1959, pp. 203–204).

This is, when described in lay terms, rather intuitive. For instance, as the amount of cognitions which justify dissonance-inducing behavior increase, the magnitude of dissonance (“D” divided by “D plus C”) is reduced. If we make a speech counter to our opinions, we must reduce the magnitude of dissonance by recruiting consonant cognitions. If we can rationalize our behavior by adding reconciliatory cognitive elements (e.g., “I got paid for this” or “I was forced to do this”), the amount of dissonance will diminish, as Festinger and Carlsmith (1959) argued. However, the same phenomenon can also be interpreted from the PP perspective, whereby the magnitude (the gain or volume) of prediction errors can be reduced by recruiting alternative hypotheses, which “explain away” the prediction error (see particularly Ch. 2 in Clark, 2016). Therefore, increasing the number of “consonant” predictions (by recruiting these higher level hypotheses) would reduce the volume of prediction error. The magnitude of dissonance, therefore, seems to be from the PP perspective particularly related to the gain or volume of prediction error units.

#### Avoidance of Dissonance

Fourth and finally, as discussed in Section “Predictive Processing: A Brief Overview,” PP and the related free energy principle propose that organisms are inclined to actively avoid error-inducing or “surprising” states. PP and “active inference” (e.g., Friston, 2010; Clark, 2016) posit that the minimization of prediction error is a driving force for organisms. This is, again, a notion which has similarities with Festinger (1957, p. 3) at its time provocative idea that CD and its active reduction are “a motivating factor in its own right,” whereby “situations and information” which would likely increase dissonance should be actively avoided to keep our cognitive models “responsive to reality.” As Clark (2016, p. 250 and 293) similarly writes, the “whole apparatus (of prediction-based processing) exists only in order to help animals achieve their goals while avoiding fatally surprising encounters with the world.” If dissonance is to be associated with prediction error, a position which I argue for in the present text, an active tendency for avoiding CD might then be understood as helping us avoid surprising bodily (proprioceptive), attitudinal/emotional (interoceptive) and environmental (exteroceptive) information flows which hinder our capabilities for achieving our goals.^7^ In Section “An Evolutionary Rationale for Cognitive Dissonance?,” this idea is elaborated further and shown in particular to be compatible with action-based models of CD.

As it appears, several similarities exist between PP and CD theory, namely, that they both deal with strikingly similar accounts of reducing unexpected (or subjectively surprising) mental states and encounters with the world. Arguably, however, PP proposes a more dynamic model of surprise reduction. From the PP point of view, dissonance reduction is not only about the modulation of “cognitive elements” in a passive stimulus–response manner, but is instead forward-looking and predictive. From this perspective, active reduction of “predictive dissonance” would serve as a primary driver for successful action in the world by means of optimizing the prediction of incoming sensory inputs. After all, the Bayesian brain proposed by PP “is an inference engine that is trying to optimize probabilistic representations of what caused its sensory input” to successfully predict the world and understand its causal mechanisms (Friston, 2010, p. 130).

Therefore, a major rationale for relocating CD in the PP framework is apparent from Festinger (1957, p. 11) following quote: “*the reality which impinges on a person will exert pressures in the direction of bringing the appropriate cognitive elements into correspondence with that reality.*” Recall Clark (2015, p. 5) critique of the standard passive and stimulus-driven cognitive model, taking “energetic inputs from the senses” and “turning them into” a coherent or consonant percept. CD, from the PP point of view, then becomes not an inconsistency between two passive elements of cognition “impinged” by a reality (as Festinger proposes), but rather as an inconsistency between active downstream (action-oriented) prediction and upstream flows of perceptual input. Dissonance reduction can therefore be reframed as the active and forward-looking reduction of conflicts between downstream predictions and upstream errors.

It seems that from this perspective, CD is strongly associable with “prediction error,” whereby CD might be useful to reframe, by performing a “Bayesian flip” of sorts (Clark, 2016), as “predictive dissonance.” This also comes an interesting response to Beauvois and Joule (1996, p. xii), who in their “radical theory of CD” note that dissonance is not a “theory of rationality, since the dissonance reduction process it describes, while clearly cognitive in nature, is also *post-behavioral* and consequently *incapable of preparing rational action.*” Importantly, from a forward-looking PP perspective, as returned to in detail in Section “An Evolutionary Rationale for Cognitive Dissonance?,” particularly the notion that dissonance is incapable of preparing rational action can be questioned. This is because if dissonance is related to, or in fact one form of, prediction error, dissonance is also pre-behavioral and thus capable of preparing efficient, successful and ecologically rational action in the long term. This might not be rational in the *homo economicus* sense (i.e., from the lens of rational choice theory), but can be generally “satisficing” (Simon, 1956, 1972). As discussed in Section “An Evolutionary Rationale for Cognitive Dissonance?,” this is not a triviality when considering the possible evolutionary origins of CD.

Predictive processing can also shed light on another confusing aspect of dissonance theory. Beauvois and Joule (1996, p. xvii) lament that the original dissonance theory by Festinger (1957) was misleading in that it led one to believe that a “dissonant relation” between cognitions always generates a “dissonant psychological state.” This is a conceptual problem which I believe can be solved efficiently with the hierarchical formulation of PP. While prediction errors, or “dissonant relations” between cognitions stem all the way up from direct sensory flow, it seems rather clear that these do not always induce dissonant psychological states. Recall that in the predictive hierarchy, all levels from highly abstract cognitions to lowest level sensory data are “accountable to the others, furnishing an internally consistent representation of sensory causes at multiple levels of description” (Friston, 2010, p. 129). This suggests that while dissonant relations could occur at multiple levels of the predictive hierarchy, ranging from direct perceptive levels (“It is raining even though it is sunny”) to more abstract identity-related levels (“I flew to an academic conference and I feel terrible about it because I have a pro-environmental identity”), what we generally refer to as the psychological discomfort of dissonance would lie at the higher end of this hierarchical model.

**FIGURE 2:**
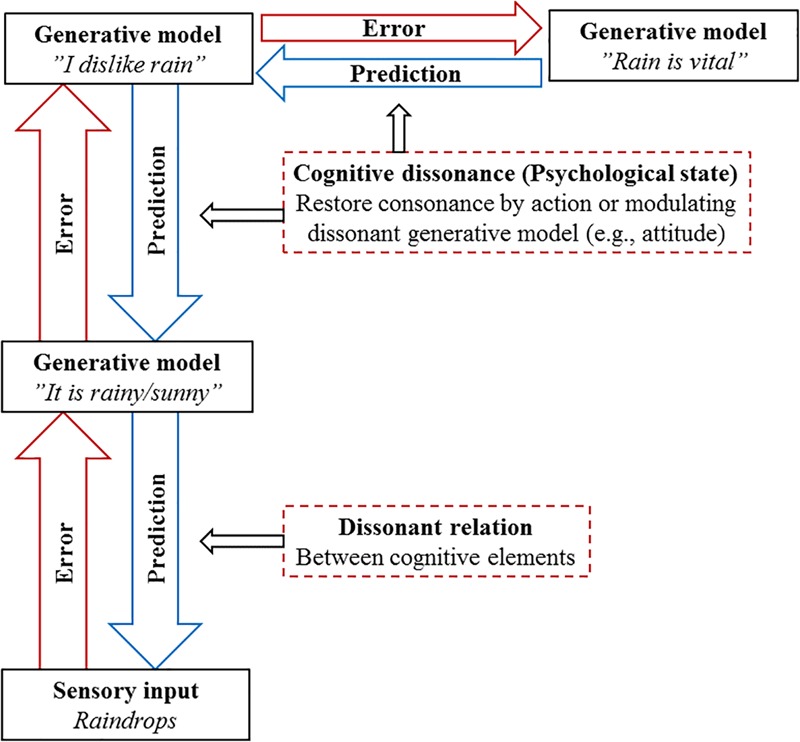
Distinguishing between dissonant relations and the psychological state of CD. While prediction error does not always induce the psychological state of CD, CD is always prediction error.

Therefore, while “dissonant relations” or “prediction errors” can exist anywhere in the hierarchical or lateral levels of the predictive hierarchy, the psychological uncomfortable state of CD (which *psychologically* motivates dissonance reduction) would most likely be those persistent and recurrent prediction errors higher in the hierarchical model. To illustrate this, imagine Festinger (1957, p. 19) picnicker from Section “Dissonance in a Nutshell” once more (see Figure 2). When the picnicker picks up the sensory information signaling the existence raindrops (i.e., sees or feels raindrops), this sends upstream prediction error to a higher-level generative model, such as “The weather is sunny and picnic-friendly.” To reduce prediction error (or the dissonant relation between cognitive elements), the generative model can be updated to “It is rainy.” I doubt, however, that this alone would induce what we call the psychological state of CD. However, we could imagine the updated generative model “It is rainy” causing further flows of prediction error to a higher-level generative model, such as “I dislike and avoid rain.” The dissonant relation between “It is rainy” and “I dislike and avoid rain” is already closer to a psychologically uncomfortable state. One could take this even further, and notice a new dissonant relation between “I dislike and avoid rain” and the recognition that “Rain is vital,” or the fact that humans and nature need rain to survive. Here, the reduction of psychological dissonance (or higher-level prediction error) could therefore be achieved by attitude change, with the picnicker finally admitting that she, in fact, highly values rainfall. In other words, the argument here is that while prediction error does not always induce the psychological state of CD, CD is always still one (high-level and persistent) form of prediction error.

Concluding this section, PP seems to have potential to be informative for the future development of CD theory. While both theories deal extensively with reductions of surprise or inconsistency (prediction error and dissonance), PP arguably gives CD valuable and informative context within the broader perception-prediction-action framework (known as active inference) and accommodates dissonance in the hierarchical multi-level system of the predictive mind. This also helps us demarcate dissonant cognitive elements from dissonant psychological states, although the two are seen as part of the same perceptual and cognitive process. In fact, the rather simple “Bayesian flip” proposed by PP, I argue below, might even help us understand why certain conservative dissonance reduction strategies exist to begin with.

## An Evolutionary Rationale for Cognitive Dissonance?

A PP take on CD can be discussed against a backdrop of research which, on the one hand, suggests that dissonance has strong evolutionary underpinnings (Egan et al., 2007), and contrarily, research which questions the compatibility between an in-born drive for dissonance reduction and human (cultural) evolution (Perlovsky, 2013). In this section, I aim to show that a theory of predictive dissonance can answer to both notions, suggesting that not only is the drive for dissonance reduction evolutionarily compatible (and even necessary), but that cultural factors can also partly define what cognitive elements are dissonant. The first proposition below is that since CD is from the PP perspective one form of “prediction error,” it is little wonder that similar processes are found from other (presumably also PP, prediction error reducing) organisms. This is an important idea to consider. In spite of long-standing interest in CD theory, its developmental or evolutionary origins are still poorly understood (Egan et al., 2007, p. 978). Second, there is nothing counter-evolutionary in reducing dissonance. On the contrary, prediction error reduction mechanisms are absolutely necessary to maintain action-readiness in a highly contingent and uncertain environment, where conservative reactions to dissonance or prediction error also prevent the overfitting of our predictive generative models to local ephemeral conditions. Below, these two “*why*s” regarding the origins of CD are discussed.

### Why #1: An Action-Based Model of Dissonance

Harmon-Jones et al. (2015) suggest an action-based model to extend the original theory of CD to respond to the question of *why* cognitive inconsistency causes both the psychological state of dissonance and the motivation for dissonance reduction. The authors’ (ibid., p. 185) proposition – drawing on influences such as William James and James J. Gibson, who also influenced the line of thought which led to at least Clark’s (2016) interpretation of PP – is that cognition exists particularly to guide behavior, and that the “negative affective state of dissonance is aroused by not all cognitive conflict but, specifically, when cognitions with *action implications* conflict with each other, making it difficult to act.” Beauvois and Joule (1996, p. 8) seem to more or less argue for a similar position, proposing that the “cognitive work of dissonance reduction is oriented by a generative cognition which is behavioral in nature” and that behavior in particular “possesses a special status in establishing the total amount of dissonance.” These ideas, again, are very compatible with the PP picture of active inference, where the predictive engine serves the fundamental purpose of guiding an organism’s behavior in a noisy and uncertain world (Friston et al., 2016). Any prediction errors inhibiting successful (and low-cost) behavior in such a world should stand corrected.

Predictive processing is particularly valuable for further understanding action-based dissonance since it proposes that predictive perceptions must be internally coherent to provide clear instructions for action. To highlight this aspect of PP, Clark (2016), pp. 33–37; drawing from Hohwy et al., 2008) uses the example of binocular rivalry. When different images (e.g., a face and a house) are presented to the right and left eye, subjects generally experience perceptual transitions from one percept (a face) to another (a house). This alternation between percepts (instead of perceiving a mixed face-house) occurs, according to the PP narrative, because no single hypothesis accounts for all the data, “so the system alternates between the two semi-stable states” (ibid.). For action-oriented purposes, this makes sense: we probably never have perceived or will perceive face-houses in our actionable environments, and our generative system has learned that “only one object can exist in the same place at the same time” (Hohwy et al., 2008, p. 691). If the opportunities for action, or affordances, of face-houses are unclear and previously unencountered (i.e., the prior probability for a face-house is low) it makes very little practical sense to perceive a face-house. Affordances here and later in the present text are defined as the relations between abilities to perceive and act and features of the environment, or in other words, the actionable opportunities afforded to a capable organism by the environment (Chemero, 2003, 2009; Kaaronen, 2017; also Gibson, 1979; Reed, 1996; Heft, 2001).

The same would arguably apply to the famous “duckrabbit,” a Gestalt demonstration by Jastrow (1899) made famous by Wittgenstein (1981). When faced with a picture simultaneously resembling the heads of both a duck and a rabbit, we do not perceive a “duckrabbit” but instead alternate between the Gestalts “duck” and “rabbit.” This makes ecological sense, since a hunter, for instance, would benefit very little from perceiving a duckrabbit, as this perception affords no coherent or consonant behavior and has not previously been encountered. A similar example, generally attributed to Kuhn (2012) and Polanyi (1958), can be made in the philosophy or psychology of science. A scientist generally does not design an experiment from the perspective of two contradictory theories. This is simply because it even though it may be analytically feasible, it makes very little pragmatic sense. As Polanyi (1958) writes, we can only encounter the scientific world successfully by “dwelling” in or embodying a theory and attending to the world from it, and one theoretical perspective is generally complex enough to satisfice for practical purposes. Predictive hypotheses, in science and in everyday life, primarily guide action, and unnecessary dissonance or error between contradictory actions should generally be eliminated to guide smooth and uninterrupted behavior.

Cisek (2007, p. 1568) “affordance competition hypothesis” can also be read to support this view. Here, Cisek argues that to “survive in a hostile environment, one must be ready to act at short notice, releasing into execution actions that are at least partially prepared,” which requires processing of “sensory information in an action-dependent manner to build representations of the potential actions which the environment currently affords.” Based on available information, these competitive affordances (or opportunities for action) are weighted against each other until a single action is selected. Selecting two contradictory actions would simply be very inefficient and impractical. Therefore, accordingly with the predictive dissonance narrative proposed in this text, any dissonant relations between competitive action hypotheses or attitudinal or intentional states pertaining to action should be reduced as quickly as possible.

A predictive dissonance account is largely in line with recent action-based models of dissonance, and has potential to be informative for the development of these theoretical frameworks. If CD is understood as one form of prediction error, it makes sense that it should be reduced to ensure successful behavior in our variant ecological niche with multiple competing affordances. As discussed in Section “Predictive Dissonance,” Festinger (1957) did seem to have this in mind when formulating the original definition of CD (where reduction of dissonance keeps us behaviorally “responsive to reality”), but this stance seemed to largely disappear from later studies, which were increasingly focused on attitudinal dissonance and less on real-world action-perception (see Cooper, 2007). Therefore, a theory of predictive dissonance seems to agree with Harmon-Jones et al. (2015) position that “dissonance processes primarily function to facilitate effective action.”

### Why #2: Overfitting Predictive Models

The other immediate “why” of dissonance relates to the statistical properties of overfitting and underfitting. Overfitting a predictive model here, as defined by Marweski et al. (2010, p. 106), would entail the generative model capturing “not only the variance due to the variables of interest but also that from random error, which organisms are likely to encounter in an uncertain world.” A predictive engine, such as the predictive mind proposed by Clark (2016), is fundamentally in the business of extracting signal from noise to prevent overfitting to a noisy, highly uncertain, and ephemeral environment. As Feldman (2013, p. 25, also in Clark, 2016, p. 272) notes, this implies separating agent-salient signal from noise to tune our priors to the world, selectively *ignoring* much of what the sensory signal makes available: “one would be unwise to fit one’s prior too closely to any finite set of observations about how the world behaves, because inevitably the observations are a mixture of reliable and ephemeral factors.”

It seems that often CD and the associated information-discarding is framed as something entirely irrational (this is particularly evident in Perlovsky, 2013). Take, for instance, the three main paradigms of dissonance research – induced-compliance (Festinger and Carlsmith, 1959), difficult-decision (Brehm, 1956), and effort-justification (Tavris and Aronson, 2008) – which all seem to focus on rather irrational aspects of dissonance, such as seemingly arbitrary attitude changes and systematic avoidance of contradictory information. However, in the ecological niche we inhabit, boundedly rational, selectively ignorant yet fast and frugal action-oriented cognition might be the most efficient way to move about and make decisions (Clark, 2016, p. 1). While perception-action of this kind is not always locally optimal, it is highly generalizable and broadly “satisficing” (Simon, 1956, 1972). This is because too much learning can lead to statistical overfitting, that is, updating the Bayesian priors of the predictive generative model to conform so closely to one set of local conditions that it is no longer generalizable to other contexts.

This might afford a new window for understanding the “origins of CD” and why dissonance occurs not only with adult humans but also other cognitive (prediction error reducing) organisms such human infants as capuchin monkeys (Egan et al., 2007). After all, any PP organism will seek to reduce long-term prediction error, so it is perhaps little surprise that cognitive phenomena such as dissonance are found in other species. The upshot is that if dissonance is related to prediction error, then it is at least not at the most fundamental level a socio-culturally acquired “learned secondary drive” (as cautiously suggested by Cooper, 2007, p. 87–89), but rather something any organism with a predictive engine will to an extent experience. The “origin” of the psychological sensation of dissonance, from the PP perspective, would then be its role in motivating prediction error reduction *in the long term*. Such a strategy implies not learning from just any discrepancies between cognitive elements, since this would be suspect to overfitting. Therefore, even when dissonance leads to a seemingly arbitrary conservation of a high-level prediction (e.g., not learning from prediction error due to self-justification or arbitrary attitude change), this does not entail that it is entirely irrational. The predictive brain, as Clark (2016, Ch. 8) amusingly writes, is after all quite “lazy,” but this might not be a fault as much as it is a necessity.

To illustrate this idea, consider a thought experiment in a social environment. Dissonance theory is, after all, originally a *social* psychological theory, so uncovering its social rationale is no triviality. Imagine here an early human called Proto, living in a primitive small culture. This culture, like any human culture, has shared meanings, intentions and norms, and the people of this culture particularly value the simple norm “Sharing is good” (you can insert practically any other simple norm here), leading this norm to be instilled at a high abstract level in their generative models. In practice, such a generative model corresponds to an increased probability of engaging in altruistic behavior, or *P(Sharing)* (since PP organisms, for better or worse, have a knack for bringing their predictions about). Now, imagine that for some reason, Proto does not share its food and, as a result, experiences cascades of CD (or, if you may, prediction error). Is it rational for Proto to update its generative model accordingly with the prediction error? Arguably not, since this would eventually lead to modulation of the high-level model “Sharing is good,” a decrease in pre-behavioral *P(Sharing)* and thus lead to all kinds of trouble in this primitive social setting (leading to, in the worst case, punishment or expulsion). Here, for instance, a more conservative self-justifying reaction to dissonance (e.g., recruitment of another cognitive element such as “I had no other choice”; “Mistakes were made, but not by me…” etc., see Tavris and Aronson, 2008) might prove to be more rational in the long run. In other words, selectively ignoring and not learning from dissonant social information might turn out to be a sound long-term strategy.

Might dissonance then be a theory of rationality after all (c.f. Beauvois and Joule, 1996)? Similar accounts of selective ignorance of information, or simple use of “fast and frugal” heuristics, are abound in the fields of bounded or ecological rationality (e.g., Simon, 1972; Marewski et al., 2010; Todd and Gigerenzer, 2012). However, the relations between ecological rationality and CD seem much less charted. From the PP perspective, CD might to at least some degree serve the purpose of motivating organisms not to overfit their predictive models to ephemeral local conditions.

This notion comes as a direct response to Perlovsky (2013, p. 1), who expresses concern regarding the compatibility between CD and human cultural evolution. According to Perlovsky (ibid.), CD inhibits acquisition of new knowledge and is thus at conflict with what fundamentally makes us human:

“This process of resolving CD by discarding contradictions is usually fast, “momentary” and according to CD theory new knowledge is discarded before its usefulness is established. This is the paradoxical conclusion of CD theory. To summarize, according to CD theory knowledge has to be devalued and discarded. But accumulation of knowledge is the hallmark of human evolution. It follows that the fact of human cultural evolution contradicts this well-established theory.”

Perlovsky (2013, p. 2) then continues: “Why have researchers of CD theory … not noticed this contradiction between its fundamental premise and the fact of human evolution?”

In the following, I attempt to respond to these questions regarding the compatibility between CD and human (cultural) evolution. First, it should be noted that Perlovsky’s account of CD is not entirely fair, since at least according to Festinger (1957) original conception, CD can lead to direct learning (a smoker might reduce dissonance by quitting the habit, and a picnicker can and often will seek shelter from rain). Yet I also argue that CD and evolution only contradict if we make the tacit assumption that more information is better for behaving successfully in the world. However, as recent advances in ecological rationality suggest (see, e.g., Marewski et al., 2010; Todd and Gigerenzer, 2012), this is not always a justifiable assumption at all, since agents in the real world “less” information is often “more.” Representing the PP perspective, I believe Van de Cruys et al. (2014, p. 1) make a valid point here: “The complex, fluctuating nature of regularities in the world and the stochastic and noisy biological system through which people experience it require that, in the real world, people not only learn from their errors but also need to (meta-)learn to sometimes ignore errors.” Perhaps the conservative reactions to CD are then exactly this kind of “meta-learning,” or in Bateson’s (1972) cybernetic terms “deutero-learning” or “second-order learning,” which help us to selectively ignore prediction errors and thus prevent overfitting. While this strategy sometimes fails miserably, in the long term it might be effective.^8^

Returning to Perlovsky (2013) concerns, it seems that they are strongly related to the so-called “darkened room puzzle” discussed in PP literature (Clark, 2016), pp. 262–265; Friston et al., 2012; Allen and Friston, 2018). The puzzle follows: Should not a “hapless prediction-driven” and error-reducing (or, for the present purpose, dissonance-reducing) organism seek out easily predicted states, “such as an empty darkened room in which to spend the remainder of its increasingly hungry, thirsty and depressing days”? Do not the “hallmarks of human evolution” (Perlovsky, 2013, p. 1) stand starkly in contrast to this? What about cultural evolution, play, exploration, and attractions of novelty (Clark, 2016, p. 262; Clark, 2017a)? The answer to the puzzle lies in the relational, cultural and contextual nature of concepts such as prediction error or dissonance. Even though it might seem like organisms such as ours might experience less “prediction error” in a dark corner in the everyday sense of the word, this would in fact be highly error-inducing, since active organisms such as humans take moving about and exploring as a baseline for predicting everyday experiences. Therefore, being stuck in an empty darkened room would be very surprising and induce high levels of prediction error – or dissonance – and we thus actively avoid such states. Thus, the PP treatment of dissonance hardly characterizes it as “A Challenge to Human Evolution,” as Perlovsky (2013) conspicuous title suggests. Rather, dissonance reduction and the associated information-discarding emerge as fundamental requirements for life in a noisy and uncertain world.

Perlovsky (2013) builds his argument to propose that music is one mechanism by which dissonance can be collectively reduced. This may (or may not) be true, but it is only one possible socio-cultural case for dissonance reduction. In fact, we humans find our entire societies structured so that we are expected to learn even contradictory information (see Ch. 9 in Clark, 2016, 2017a). Take, for instance, schools and universities, where students are assumed to challenge their folk understanding of physics, society, or psychology. If an undergraduate student, for instance, is socially and culturally expected to learn information which is contradictory to their prior knowledge, an incapability for learning such knowledge would likely result in the highest magnitude of dissonance (i.e., much higher than the contradictory information causes by itself). Reduction of dissonance could hereby, in some social settings, simply entail learning. Socio-cultural scaffoldings, from the PP perspective, therefore serve as collective bootstraps which instill to our predictive minds the norms of learning and exploring (Clark, 2017a), even when this information is dissonant with prior cognitions. This is quite a different picture from Festinger (1957, p. 20) claim that “the possibilities of manipulating the environment [to reduce dissonance] are limited.” From a socio-cultural PP perspective (see Clark, 2016; Ramstead et al., 2016), environment manipulation and niche construction (Constant et al., 2018) can be interpreted as being fundamental to dissonance reduction and consequent meta-learning patterns. We live, as Clark (2016) calls it, in “bootstrap heaven,” where a “ratchet-effect” (Tomasello, 1999) of socio-cultural processes takes place to create new environments with novel expectations – this also alters what we experience as “prediction error” or “CD,” which appear to be highly culture-dependent concepts (Clark, 2017a).

The long-term weighting of prediction errors is a delicate business, as updating the Bayesian priors of our predictive engines can (and, in practice, do) result both in over- and underfitted generative models. Dissonance, it seems, might fundamentally be related to the prevention of overfitting, although the side-effect of this is obvious. Dissonance reduction *via* selective sampling, for instance, might also cause drastically underfitted generative models, leading to persistent confirmation biases (Clark, 2016). But this is the price, if you may, of the generalizable, ecologically rational, and action-enabling predictive dynamic. An active species such as ours, after all, operates in a “rich landscape of affordances,” or an ecological niche with spectacularly rich and variant opportunities for action (Rietveld and Kiverstein, 2014). A rich affordance landscape, in turn, requires a highly generalizable and efficient predictive model. That, at least for our species, seems to have been phylogenetically satisficing (this is, in fact, self-evidenced by our very existence, see Friston, 2018). However, as discussed briefly below in Section “Underfitted Generative Models and Ecological Crises,” this strategy of overfitting-prevention is causing all kinds of trouble in a 21st century context, where our ecological niches and landscapes of affordances are changing more rapidly than our predictive capabilities.

## Underfitted Generative Models and Ecological Crises

I end with a normative note. CD is arguably one of the more relevant cognitive phenomena in understanding the roots of the global ecological crisis. This is due to the apparently prominent role that CD plays in the so-called attitude–action gap in environmental psychology (see Kollmuss and Agyeman, 2002; Kaaronen, 2017; Uren et al., 2018) as well as climate denialism and other ecological “denialisms” (Lorenzoni et al., 2007). Here, it seems, dissonance arising between generative models and sensory input (e.g., dissonance between attitudes and action) is explained away by mechanisms such as delegation of responsibility, distancing, apathy, denial, and active avoidance of contradictory information (Stoll-Kleemann et al., 2001; Kollmuss and Agyeman, 2002). Simply, we are not updating our predictive generative models to adequately respond to the urgency at hand. Therefore, studying CD in this day and age might be more important than ever before.

Phenomena such as climate denialism might be understood better through the PP viewpoint, which would posit that strong priors (i.e., mental models attuned to environmentally harmful behavioral habits) combined with low attention and selective sampling are at least partly to blame for the poor uptake of new behaviors and evidence-informed local and global policies. The latter case is particularly interesting, since while science already deals with separating noise from signal, subjectively perceived sensory inputs (e.g., from science communication) might still be treated as any other noise by a layperson’s predictive model (see Stoll-Kleemann et al., 2001 and Lorenzoni et al., 2007 for relevant empirical cases). This, one could argue, results in radical underfitting, and many typical reactions to climate dissonance – such as self-justification, avoidance of contradictory information, or delegation of responsibility – do not nearly as much prevent overfitting as they induce systematic underfitting, risking the future minimization of prediction error, and thus human existence. Our locally tuned and “fast and frugal” predictive minds, it seems, are not particularly well suited for dealing with the prediction of slow, abstract, and global non-linear phenomena, such as ecocide, the sixth mass extinction, and climate change, which would only induce *unavoidable* high-volume prediction error when irreversible ecological tipping-points^9^ are reached, or when it is too late.

Perhaps a further developed theory of predictive dissonance could inform new models of environmental education, communication and behavioral policies, which would focus on ways to induce salience-based error signal enhancement (i.e., “boosting” relevant error signals). I have suggested elsewhere that a necessary starting point for this would be the design of urban environments, urban affordances and environmental education which promote local “action-first” environmentalism, by increasing attention toward environmental concerns *via* structurally induced behavior (Kaaronen, 2017). Undeniably, however, much further research and experimentation is required. PP presents an interesting opportunity here, by employing cutting-edge understanding of the mechanisms of the human mind to help us in the “art of the cognitive war to save the planet” (Antal and Hukkinen, 2010).

## Conclusion

I have argued in this article that PP (also predictive coding) can help us further understand the theory of CD, and that the theory of CD could be accommodated within the broader PP framework. The PP take on CD, in this article, has been dubbed “predictive dissonance.” This owes to the notion that it seems like CD is strongly related to the concept of prediction error in PP. Conclusively, PP can be interpreted to shed further light on:

• *What* CD is. From the PP perspective, dissonance arises from upstream error signals weighed variably against downstream predictive generative models. This can also be dubbed “predictive dissonance.” The magnitude of dissonance is a function of the gain or volume and persistence or recurrence of error signals. Predictive dissonance can be reduced by either updating the predictive model, recruiting alternative predictive models or changing behavior and moving about in the social and material world (altering the harvested information flow).
• *Where* CD exists. From the PP perspective dissonance can occur at different hierarchical (or lateral) levels in the multi-level predictive hierarchy. This, at least partly, solves the dilemma of whether “dissonant relations” between cognitions always generate a “dissonant psychological state” (Beauvois and Joule, 1996). A theory of predictive dissonance proposes that while “dissonant relations” or “prediction errors” can exist anywhere in the hierarchical or lateral levels of the predictive hierarchy, a psychological uncomfortable state of CD would most likely be those persistent prediction errors higher in the predictive hierarchy.
• *Why* CD exists to begin with. Egan et al. (2007, p. 982) speculate whether there “may be some core aspects of cognition that give rise to CD” to enlighten its evolutionary origins and, indeed, such a mechanism might be explained by a theory of predictive dissonance. As discussed in Section “An Evolutionary Rationale for Cognitive Dissonance?,” conservative responses to dissonance could exist at least partly to prevent the overfitting of predictive models to local and ephemeral conditions, since this would lead to poorly generalizable predictions. From this perspective, dissonance is not “post-behavioral” or “incapable of preparing rational action” (c.f. Beauvois and Joule, 1996), but also pre-behavioral and (often, but not always) capable of preparing ecologically rational, fast and frugal, action. Dissonance then, is very action-oriented indeed – a notion which resonates well with the work of Harmon-Jones et al. (2015). Any strategy which prevents overfitting, however, is suspect to underfitting (or not learning from prediction errors from which one ought to learn), which might propagate some ecologically unsound cognitive phenomena such as climate denialism and similar biases.

A picture of CD is therefore painted where perception and action are self-fulfilling prophecies, and “When Prophecy Fails” (to evoke the title of Festinger et al., 1956), we experience varying magnitudes of dissonance. CD has helped and still does help organisms such as ours to “surf the waves of uncertainty” (Clark, 2016), but in today’s world, this seems to often happen at a dire ecological and social cost. Since CD is presumably here to stay, we need to bring it up to date with the most recent advances in cognitive sciences. I have proposed in this paper that CD can be accommodated in the broader cognitive theory of PP. PP is a hastily and widely propagating theory, with new developments in a variety of fields ranging from machine learning to psychopathology, and keeping dissonance theory up to date with these advances and even their computational and experimental methodologies could pave a fruitful way forward.

## Author Contributions

RK made sole contribution to the conception and design of the work; drafted the work and revised it critically for important intellectual content; and agreed to be accountable for all aspects of the work in ensuring that questions related to the accuracy or integrity of any part of the work are appropriately investigated and resolved.

## Conflict of Interest Statement

The author declares that the research was conducted in the absence of any commercial or financial relationships that could be construed as a potential conflict of interest.

## Footnotes

1 See, e.g., Friston (2010) or Seth (2014) for concise accounts of the “Bayesian brain” hypothesis.

2 Priors can here be defined as the probability distributions or densities “of the causes of data that encodes beliefs about those causes before observing the data” (Friston, 2010, p. 129).

3 In PP literature, it is common to find descriptions of predictions and prediction errors flowing hierarchically (downstream and upstream). However, as Clark (2016, p. 308) notes, this is just a shorthand, since “a great deal of predictive information is also passed laterally within levels.”

4 Of course, these forward-looking and action-oriented views of the mind are far from new. As Clark (2016, p. 289) notes, such ideas can be readily found in the phenomenology of Merleau-Ponty (1962), although one could as justifiably add pragmatists such as Dewey (1896) and James (1884) to the mix (next to obvious enactivists in the vein of, e.g., Varela et al., 1991). A less known similar theory of perception can also be found in Michael Polanyi (1965; 1958) “tacit inference” (or “from-to” structure of knowledge), where perception occurs from embodied “tacit knowledge” to a target of perception, which is perceived in light of embodied (prior) tacit knowledge. Polanyi (1965, p. 2) tacit inference is action-oriented and predictive: “When we perform a skill, we attend focally to its outcome, while being aware subsidiarily of the several moves we co-ordinate to this effect.” What these otherwise greatly intriguing theories of perception-action seem to lack is a coherent account of the mechanism of how (and why) these “tacit” or embodied predictive priors are updated.

5 This is similar to Conant and Ross Ashby (1970, p. 89) cybernetic discussion on what makes a “good regulator”: “the living brain, so far as it is to be successful and efficient as a regulator for survival, *must* proceed, in learning, by the formation of a model (or models) of its environment.”

6 The reader is directed to Odling-Smee et al. (2003) or Constant et al. (2018) for more detailed discussion on niche construction.

7 These stimuli are defined as follows (see Clark, 2016): Interoceptive: stimuli produced within an organism, which include emotional and attitudinal states. Proprioceptive: stimuli relating to behavior, bodily movement, and position. Exteroceptive: stimuli picked up from the surrounding external environment.

8 As an interesting and relevant side note, locally overfitted generative models, which weigh some prediction errors significantly higher than the neurotypical population, have been associated with traits found in the autism spectrum. Such strategies result in overwhelming prediction errors and seem to lead to the enforcement of predictable, and arguably less broadly adaptable, routines (Van de Cruys et al., 2013, p. 96, also in Clark, 2016, p. 226).

9 Tipping points are situations where an ecosystem experiences abrupt and often self-reinforcing transitions to new distinct states, with significant implications for climate, biodiversity, and other ecological and social systems (Barnosky and Hadly, 2015).
